# Oxygenation, Ventilation, and Airway Management in Out-of-Hospital Cardiac Arrest: A Review

**DOI:** 10.1155/2014/376871

**Published:** 2014-03-03

**Authors:** Tomas Henlin, Pavel Michalek, Tomas Tyll, John D. Hinds, Milos Dobias

**Affiliations:** ^1^Department of Anaesthesia and Intensive Medicine, Military University Hospital, 1st Medical Faculty, Charles University in Prague, 160 00 Prague, Czech Republic; ^2^Department of Anaesthesia and Intensive Medicine, General University Hospital, 1st Medical Faculty, Charles University in Prague, 120 21 Prague, Czech Republic; ^3^Department of Anaesthesia, Craigavon Area Hospital, BT63 5QQ Portadown, Northern Ireland, UK

## Abstract

Recently published evidence has challenged some protocols related to oxygenation, ventilation, and airway management for out-of-hospital cardiac arrest. Interrupting chest compressions to attempt airway intervention in the early stages of OHCA in adults may worsen patient outcomes. The change of BLS algorithms from ABC to CAB was recommended by the AHA in 2010. Passive insufflation of oxygen into a patent airway may provide oxygenation in the early stages of cardiac arrest. Various alternatives to tracheal intubation or bag-mask ventilation have been trialled for prehospital airway management. Simple methods of airway management are associated with similar outcomes as tracheal intubation in patients with OHCA. The insertion of a laryngeal mask airway is probably associated with worse neurologically intact survival rates in comparison with other methods of airway management. Hyperoxemia following OHCA may have a deleterious effect on the neurological recovery of patients. Extracorporeal oxygenation techniques have been utilized by specialized centers, though their use in OHCA remains controversial. Chest hyperinflation and positive airway pressure may have a negative impact on hemodynamics during resuscitation and should be avoided. Dyscarbia in the postresuscitation period is relatively common, mainly in association with therapeutic hypothermia, and may worsen neurological outcome.

## 1. Introduction

Since the late 1950s, when Safar et al. described the ABC principle in cardiopulmonary resuscitation [1, 2], the letters “A” (airway) and “B” (breathing, ventilation) have been the cornerstones of resuscitation in cardiac arrest. For many years, this algorithm remained unchanged. Opening the airway, delivering oxygen at 100% concentration, insertion of a tracheal tube, and application of intermittent positive pressure ventilation (IPPV) were considered “gold standards” in oxygenation and airway management. This applied both during cardiopulmonary resuscitation for cardiac arrest in adults and also in the early period after restoration of spontaneous circulation (ROSC). However, the outcomes of patients after out-of-hospital cardiac arrest (OHCA) remained quite poor. In the United States, survival rate to hospital admission is 26.3%, and only 9.6% of patients are able to be discharged from inpatient care [3].

Many CPR standards have been challenged during the last decade in adult cardiac arrest of nontraumatic origin. This has included the method of delivering oxygen, its ideal fraction, ventilation strategies, timing, and utilizing adjuncts other than a tracheal tube for maintenance of airway patency.

## 2. Management during Resuscitation

### 2.1. Oxygenation in Cardiac Arrest

Oxygen requirements in cardiac arrest and in the period after return of spontaneous circulation (ROSC) have been extensively studied during recent years. Maximizing oxygen delivery (DO_2_) is paramount during the period of cardiac arrest and ineffective circulation for aerobic metabolism and synthesis of adenosine triphosphate (ATP) [4, 5]. High paO_2_ does not cause intracellular or tissue hyperoxia at this time. The consensus is that, during cardiac arrest, 100% oxygen should be delivered to victims in order to increase arterial and tissue pO_2_ [6, 7]. Debate continues as to whether oxygen should be delivered via bag-mask ventilation, tracheal tube, supraglottic airway devices or via passive oxygenation [8, 9]. What is more controversial is the most appropriate oxygen fraction (FiO_2_) to deliver once restoration of spontaneous circulation has been achieved [10]. Oxygenation strategies in the post-ROSC period are described in detail in another section of this paper.

Novel and alternative oxygenation methods and strategies in adult out-of-hospital cardiac arrest are discussed in following paragraphs.

### 2.2. Concept of Passive Oxygenation

The concept of continuous passive flow of oxygen to the airway was developed on animal models (dogs) in 1982 [11]. Same authors showed that anesthetized and paralyzed dogs may be oxygenated using this method for a relatively long period [12]. Passive oxygenation was first described in humans in 1991 [13]. Brochard and colleagues used specially equipped tracheal tubes with inserted microcannulas which allowed delivery of a constant flow of concentrated oxygen in ICU patients during disconnections of their breathing circuit. This study was followed by Saïssy et al. who evaluated passive insufflation of oxygen in adult patients during cardiac arrest outside the healthcare facilities [14]. The design of this study was prospective, randomized, and controlled. In total, 48 persons were managed using passive oxygenation, while, in the control group, another 47 patients were ventilated with intermittent positive pressure ventilation. There were no differences in the main outcomes studied—percentage of patients with ROSC or number of victims surviving until hospital admission.

Unfortunately, the neurological outcome of resuscitated individuals was not reported. A subsequent large prospective randomized trial evaluated 1,042 patients with OHCA, assigned to receive either conventional mechanical ventilation or constant flow insufflation of oxygen (CFIO) [15]. The authors did not find any difference in ROSC rates, admissions to hospital, or successful discharge from intensive care facilities. The ICU discharge rate was very low in both groups (2.3% conventional ventilation versus 2.4% in CFIO patients). These two studies used passive oxygen insufflation through a modified tracheal tube (Boussignac tube).

Different results were reported by a group from Arizona. In their first study, Bobrow et al. retrospectively analyzed 1,019 patients who were managed during resuscitation either with positive pressure bag-mask ventilation or with passive insufflation of oxygen through an oropharyngeal airway [16]. Significantly higher survival without neurological deficit was found in the passive oxygen insufflation subgroup—38.2% versus 25.8%, though only in witnessed VF/VT arrest. No difference in outcomes was noted in this study for unwitnessed VF/VT arrest patients or for cardiac arrests caused by nonshockable rhythms. The same group of researchers evaluated in total 4415 scenarios of OHCA in adults caused by a heart disease during a 5-year period [17]. Persons in this study were found by lay bystanders. They were divided into three groups according to the mode of CPR—conventional CPR with chest compressions and mouth-to-mouth breathing, chest compression-only CPR (COCPR), and no CPR provided on scene. COCPR group had the highest survival to discharge from hospital—13.3%. While the results of previous studies suggested a beneficial role of passive oxygen insufflation, the latest trial of Bobrow et al. [17] and results of other studies [18] suggested that the main advantage of this mode of resuscitation—COCPR or cardiocerebral resuscitation (CCR)—is probably through the constant delivery of chest compressions, without interruptions for advanced airway interventions, than the passive application of oxygen “per se.” Therefore, cardiocerebral resuscitation is accepted in the early phases of OHCA of cardiac origin [19]. Several animal studies have reported the usefulness of CFIO in the early stages of cardiac arrest compared with conventional ventilation, but their interpolation into human medicine is problematic [20, 21]. Passive insufflation of oxygen is probably not sufficient for an adequate gas exchange during advanced stages of cardiac arrest when chest resistance is higher and lung compliance significantly decreases [22].

Both ERC and AHA guidelines mention passive oxygen delivery in their recent guidelines [6, 7] but do not recommend its routine use during cardiopulmonary resuscitation until more clinical data become available.

### 2.3. Airway Management Strategies

Management of the patent airway during OHCA may be divided into basic and advanced. Basic airway management consists of the manual relieving of upper airway obstruction (“triple maneuver”), bag-valve mask ventilation (BMV), or the insertion of oropharyngeal or nasopharyngeal airway [6, 7]. Techniques of advanced airway management include the insertion of a supraglottic airway device (SAD) [23], tracheal intubation [24], insertion of Combitube [25], or cricothyrotomy [26]. For many years, all resuscitation algorithms and protocols recommended early tracheal intubation as a part of prehospital advanced life support (ALS). Arguments favoring early tracheal intubation mainly revolved around expectations for better control of the airway, protection against upper airway obstruction, decreased risk for aspiration of gastric contents, and better control of carbon dioxide removal [25]. The strategy of airway management in OHCA in adult patients has gradually shifted towards less invasive techniques during the last decade. ERC guidelines from 2010 recommend performing prehospital tracheal intubation only if a competent intubator is present at the site of OHCA, and with only minimal interruption of chest compressions [6, 8]. AHA guidelines recommend using the most familiar device for the rescuer and conclude that an insertion of supraglottic airway device may be an equivalent to bag-mask ventilation or tracheal intubation [7].

#### 2.3.1. Tracheal Intubation

The major concerns associated with prehospital tracheal intubation in OHCA include a low success rate, long duration of intubation attempts with interruption of chest compressions, and unrecognized tube misplacement or inadvertent esophageal insertion [27]. The total success rate of prehospital tracheal intubation performed by nonphysicians varies between 75 and 90% [26–28]. Jones and colleagues found that 5.8% of all patients intubated outside hospital had their tracheal tube outside the trachea [29]. Bair et al. reported a 2% incidence of incorrect positioning of the tracheal tube at admission to hospital, unrecognized by paramedics [30]. Other authors reported an even higher incidence of tracheal tube malpositioning—6.7% of esophageal intubation and 10.7% of endobronchial intubation [31]. The correct positioning of the tracheal tube inside the trachea should be always confirmed by an etCO_2_ detection device. An esophageal detector device can be used to avoid esophageal placement [32]. Wang et al. evaluated the impact of intubation errors in the out-of-hospital setting on patient outcome [33]. One or more errors were reported in 22.7% of patients (failed tracheal intubation in 15%, multiple attempts in 3%, and tube malpositioning also in 3%). However, these errors were not directly linked to increased mortality. Difficulties with, and failures of, tracheal intubation in the prehospital environment may be caused by conditions which are often far from ideal—too little or too much light, patient position, and lack of space—and exacerbated by the low exposure of many paramedics to regular tracheal intubation. The average incidence of tracheal intubation performed by individual EMS providers is estimated at between 1 and 4 per annum [27, 34]. The incidence of difficult intubation in prehospital medicine is over 10%, with independent contributing factors being obstructed airway, intubation on the floor, and a distance between hyoid bone and tip of the chin less than 4.5 cm [35].

Attempts for tracheal intubation may cause significant interruption to chest compressions during CPR for OHCA. The median duration of interruptions caused by tracheal intubation was 109.5 s, with more than one-third of patients requiring more than two attempts for successful tracheal intubation [36]. Another study evaluated the number of attempts needed for successful tracheal tube placement in the prehospital setting [37]. More than one attempt was required in more than 30% of patients. Cumulative success rate in OHCA for the first three intubation attempts was 69.9%, 84.9%, and 89.9%, respectively. However, the success rate for tracheal intubation was significantly higher in OHCA patients than in the scenario of nonarrested subjects requiring sedation. Egly et al. studied the influence of prehospital intubation on survival of patients with OHCA [38]. Retrospective analysis included 1515 cases of OHCA. Patients with ventricular fibrillation or ventricular tachycardia who were intubated showed lower survival rate to discharge while, in the whole cohort, there was no difference found between intubated and nonintubated subjects.

Some countries, as Germany, Austria, or the Czech Republic, have physicians trained in anesthesia or emergency medicine available as part of a coordinated prehospital ambulance service response. The risks associated with tracheal intubation amongst these services are therefore lower, with the first pass and overall success rates being higher. Under these conditions, prehospital tracheal intubation may offer a benefit over other methods [39].

#### 2.3.2. Supraglottic Airway Devices

Several studies have compared the insertion of a supraglottic airway device (SAD) with conventional tracheal intubation in cardiopulmonary resuscitation in the adult population. Percieved benefits of an alternative airway management using an SAD in cardiac arrest include a shorter time of device insertion and higher success rates than tracheal intubation when performed by paramedics and other nonanesthesiologists [23]. Most published studies are nonrandomized mainly due to ethical reasons.

Tanabe and colleagues performed a nation-based study of 318141 patients with OHCA [40]. Advanced airway management techniques were used in 43.5% and included esophageal obturator (63%), laryngeal mask airway (25%), and tracheal tube (12%). Both SADs were associated with significantly worse neurological outcome than tracheal intubation. Kajino et al. studied the influence of airway management technique on outcome in OHCA using a prospective cohort design [41]. In total, 5377 cases received advanced airway management following cardiac arrest (31.2% using tracheal intubation, 68.9% using a supraglottic airway device). There were no differences either in survival or incidence of good neurological outcome between devices, although tracheal intubation took a significantly longer time. Shin and colleagues studied the outcome of 5278 patients with OHCA whose airways were managed using bag-mask ventilation, tracheal intubation, or laryngeal mask airway [42]. The latter option showed the lowest rates of survival to hospital admission and also reduced survival to discharge from hospital. The main limitation of the study was the significant disproportion between the airway management techniques used (BMV 87.9%, TI 7.4%, and LMA 4.7%). Similar results were published by Wang et al. who performed a secondary analysis of data related to airway management from ROC PRIMED trial [43, 44]. Successful tracheal intubation was associated with better early survival and higher hospital discharge rates when compared to insertion of an SAD during OHCA [43].

It is appropriate to mention some limitations of SAD use during CPR for cardiac arrest. Laryngospasm is sometimes present in the early stages of cardiac arrest as a protective airway reflex against aspiration. Higher peak inspiratory pressures are necessary to overcome laryngospasm and may exceed the maximal seal pressure of the SAD device, causing a significant leak or ineffective ventilation [23]. During elective surgical procedures under general anesthesia major leak is seen only in 0–5% of cases [45, 46] while, during CPR, it may reach more than 20% [47, 48]. SADs are also ineffective in providing controlled ventilation in patients with very low chest compliance and high rigidity, as seen in drowning persons or in the advanced stages of cardiac arrest [49]. SADs furthermore provide only limited protection against aspiration of gastric fluid and very low protection against aspiration of solid gastric contents. However, most patients with OHCA aspirate before arrival of the EMS and before attempts for advanced airway management [50]. The 2nd generation SADs such as the ProSeal LMA, Supreme LMA, and i-gel supraglottic airway [51] should theoretically provide better protection against aspiration of gastric contents. Insertion of the i-gel and Supreme LMA seems to be easier than with the LMA Classic [23]. The i-gel resuscitation pack has been developed specially for CPR scenarios and incorporates a side channel for passive delivery of oxygen [52], but clinical experience is so far very limited [53]. The i-gel airway has showed 100% insertion success rate in OHCA with 97% of patients receiving effective ventilation. Furthermore, insertion of the device did not cause any interruptions in chest compressions in 74% of victims [48]. Other SADs trialled in OHCA included laryngeal tube (85.3% insertion success rate), which was not considered to be an appropriate adjunct in CPR due to high incidence of failure and other complications [47], intubating LMA, LMA Supreme, LMA ProSeal, and CobraPLA. The Combitube has been trialled for prehospital airway management mainly in the United States. Wang et al. in their paper reported 1521 Combitube insertions in out-of-hospital scenarios (1.7% of all airway interventions) [27]. The Combitube has an overall insertion success rate almost 98% but its use may be associated with serious complications including esophageal perforation or airway trauma and has proven difficult to insert in people with neck immobilization with a cervical collar [54].

In conclusion, the laryngeal mask airways and the i-gel might be considered as alternate airway devices in OHCA. Other SADs have lower success rate or carry a higher risk of potentially serious complications.

#### 2.3.3. Bag-Valve Mask Ventilation

Bag-valve mask ventilation (BMV) is a fundamental basic airway skill. During its application with a self-inflating bag, maintenance of a patent upper airway is mandatory. This can be achieved with a “triple maneuver” (jaw thrust and neck extension) or with the insertion of an oropharyngeal or nasopharyngeal airway [25]. BMV is an easy method, often applicable without difficulty by paramedics or even by laypersons. Using intermittent positive pressure ventilation, the main adverse effects associated with BMV are stomach distension, airway leak (up to 40%), and lack of protection of the airway against aspiration [50]. Regurgitation may occur in 12.4% of patients ventilated with BMV during OHCA while insertion of LMA may decrease this risk to 3.5% [50]. Another study showed an even higher incidence of this complication—20% of patients regurgitated at the scene and 24% of all resuscitated persons had radiological findings of aspiration on chest X-ray after admission to hospital [55]. All patients were intubated at the scene. A prospective population-based study (All-Japan Utstein Registry) evaluated 649,359 patients with OHCA [56]. Primary outcome of this trial was neurological outcome related to different airway management technique during CPR and prehospital emergency care after ROSC. In total, 57% of patients were managed using bag-mask valve ventilation while 37% of them had inserted a supraglottic airway device and only 6% underwent prehospital tracheal intubation. BMV was associated with a significantly higher chance for neurologically favorable outcome than tracheal intubation or supraglottic airway device insertion. No difference in terms of neurologically intact survival was reported between patients receiving tracheal intubation or a supraglottic airway device.

In another smaller study, a group of patients with OHCA managed using BMV showed a comparable rate of survival without neurological deficit compared to patients who underwent prehospital tracheal intubation [57].

### 2.4. Extracorporeal Oxygenation and Life Support

The term extracorporeal cardiopulmonary resuscitation refers mainly to the technique of venoarterial extracorporeal membrane oxygenation (VA-ECMO) [58]. This technique may be indicated in both out-of-hospital and in-hospital cardiac arrests, mainly those refractory to conventional CPR. Venous blood is led to a membrane oxygenator and then oxygenated blood returned to the arterial circulation of the victim. VA-ECMO is used as a bridging therapy in arrested patients with severely impaired ventricular function, until either their heart function improves or before utilization of a mechanical ventricular assist device [59]. The main prerequisite for the use of the VA-ECMO in cardiac arrest is undamaged or only minimally affected brain function [60]. The use of cardiopulmonary bypass in prolonged cardiac arrest was firstly described by Safar et al. in 1990 [61]. Extracorporeal support devices have undergone significant technological advances over the years in terms of simplicity, portability, and miniaturization.

Several studies have explored the efficacy of VA-ECMO in cardiac arrest in terms of mortality and neurological outcome. An initial report described up to 20% survival rate after in-hospital cardiac arrest of adult patients [62].

Three-month neurological outcome following CPR for nontraumatic cardiac arrest was evaluated in a cohort of 162 adult patients [63]. VA-ECMO was initiated in 53 patients while conventional CPR was used in the remaining 109 victims. Survival with neurologically unchanged brain function was significantly higher in the VA-ECMO group—29.2% versus 8.3% (*P* = 0.018). The only independent predictor associated with a favorable neurological outcome at 90 days was the diameter of the victim's pupils at time of hospital admission.

Reports evaluating the efficacy of extracorporeal CPR in adult OHCA were appraised in an article by Morimura et al [60]. The authors collected a sample of 1282 victims (from 105 articles) who received the VA-ECMO during CPR. The overall survival rate, including discharge from hospital, was 26.7%. Most surviving patients presented as neurologically intact or having a mild disability only.

Chen and colleagues performed a three-year prospective observational trial assessing the efficacy of VA-ECMO versus conventional CPR in witnessed in-hospital cardiac arrest [64]. They found a significantly higher 30-day survival rate, discharge rate from hospital, and 1-year survival rate in the extracorporeal support group.

Most centers have reported significantly lower survival of patients with out-of-hospital cardiac arrest treated with the VA-ECMO when compared with patients who had witnessed cardiac arrest of cardiac origin in hospital [65, 66].

Le Guen et al. in their study reported very low survival rates (4%) in patients supported with VA-ECMO following OHCA and recommended a rather restricted approach for its use for this indication [67].

ERC guidelines recommend consideration of extracorporeal life support in various scenarios, but not in out-of-hospital cardiac arrest of cardiac origin [6, 8]. AHA 2010 guidelines do not recommend extracorporeal life support techniques for routine use in patients with cardiac arrest. The use of extracorporeal techniques should be considered only in specialized centers and in persons with a good chance for neurological recovery [68].

### 2.5. Hyperventilation and the Effect of Positive Airway Pressure

Hyperventilation and intermittent positive pressure ventilation (IPPV) “per se” have negative effects on circulation during CPR and after ROSC [69]. Positive airway and intrathoracic pressures during the mechanical inspiration phase of the breathing cycle cause a significant decrease in venous return to the thoracic cavity, reducing preload to the right heart [70]. Hyperinflation results not only in a fall in cardiac output and performance of the right ventricle, but also cause a significant reduction in coronary perfusion pressure [69] enhancing hypotension [71]. In published studies, most paramedics ventilated patients at a higher frequency and at higher inspiratory pressures than recommended [72]. A special device—impedance threshold device (ITD)—has been developed in order to reduce intrathoracic pressures, with a resulting improvement in venous return and coronary blood flow during CPR [73, 74]. A valve inside the ITD closes during chest wall recoil and helps to create a negative intrathoracic pressure as low as −13 mmHg [75]. Various clinical studies have assessed the effect of ITD on survival and neurological outcome after OHCA. In total, seven randomized controlled trials have assessed the ITD in prehospital emergency care. A study by Plaisance et al. showed better coronary perfusion and higher diastolic pressures in patients treated with ITD valve [76] while another trial compared the use of ITD with a sham device during CPR in twenty-two patients with OHCA and showed marked improvement in systolic pressure in the ITD group [77]. Use of the ITD combined with active compression-decompression was associated with increased hospital admission and short-term survival rates [78, 79]. Aufderheide et al. compared ITD with a sham device during standard CPR in 230 patients [71]. The subgroup of people who presented with a pulseless electrical activity (PEA) showed higher 24 h survival, while there was no difference in patients with VF or asystole. A meta-analysis based on available trials [80] concluded that the use of ITD may improve short-term outcome after OHCA. None of the studies evaluated hospital discharge rate in terms of neurological deficit. A robust multinational study unfortunately did not confirm the conclusions of this meta-analysis [81]. The authors evaluated 8,718 patients with OHCA randomly allocated to an active treatment with ITD and to sham group and found no differences in survival, ROSC, or recovery without neurological dysfunction. Similar concerns were also reported by some animal studies. These trials reported either no positive effect of ITD [82, 83] or indeed a worse outcome in the ITD groups [84].

On the other hand, if an ITD is combined with active compression-decompression it improves hospital discharge with neurologically favourable outcome when compared with conventional CPR [78].

The real significance of clinical studies assessing the role of ITD in OHCA might be confused by the fact that some studies compared ITD use with conventional CPR only, whilst other studies implemented ITD application with the use of active compression-decompression CPR [81].

ERC guidelines do not recommend the routine use of the ITD due to a lack of data confirming its benefit in long-term survival of victims [6, 7]. AHA guidelines recommend consideration of ITD use by the staff familiar with the device during OHCA (level of evidence B, class IIb) [68].

## 3. Management after Resuscitation (Post-ROSC Period)

### 3.1. Hyperoxemia after Resuscitation

Hypoxemia has deleterious and potentially lethal effects on vitally important organs, mainly on the brain and myocardium. However, recent studies have shown that hyperoxemia may also have significant negative effects in the postcardiac arrest period, primarily on neurological outcome [10, 85]. Excessive oxygen is a precursor for reactive forms of oxygen (reactive oxygen species—ROS, oxygen free radicals—OFR) which are created after restoration of spontaneous circulation in the tissues as a part of ischemia-reperfusion injury [5]. Mainly superoxide, hydroxyl radicals, and peroxynitrite cause direct damage to the cells which may result in their worsened function or death.

Several animal trials and data from three human studies support this theory. The effect of different oxygen fraction on neurological outcome after experimental cardiac arrest in animals was firstly evaluated by Balan et al. 2006 [85]. The authors induced ventricular fibrillation in 17 dogs and then resuscitated them using open-chest CPR. The dogs were subsequently randomized to receive either 100% O_2_ IPPV or controlled ventilation with FiO_2_ adjusted according to pulse oximetry measurements (target spO_2_ was 96%). In the hyperoxemic subgroup paO_2_ rose to 75.2 (±4.8) kPa while, in the oximetry subgroup, it remained within the physiological range—12.5 (±0.5) kPa. Hyperoxemic dogs showed a higher incidence of neurological deficit at 23 hours, as well as a higher number of pathologically altered neuronal changes in the CA1 region of the dorsal hippocampus. Similar findings were also reported by Liu et al, who demonstrated better neurological recovery and a lower degree of lipid oxygenation in the brain at 24 h on a canine model of ventricular fibrillation in normoxemic animals (FiO_2_ 0.21) than in a hyperoxemic group (FiO_2_ 1.0) [86]. Vereczki et al. performed another study on a canine model of cardiac arrest and demonstrated that normoxemic animals in the post-ROSC period had a lower level of oxidative stress, decreased intraneuronal protein nitration, and a lesser extent of neuronal death in the hippocampus [87]. Another study performed on swine model ventilated with 100% oxygen for 60 min after ROSC also showed a significantly higher degree of degeneration of neural cells in the striatum when compared with the group ventilated with FiO_2_ 0.21 10 minutes after ROSC [88]. These findings are, however, disputable because of the retrospective study design and insufficient number of probands.

Angelos et al. demonstrated a deleterious effect of post-ROSC hyperoxemia on a rat model. Sprague-Dawley rats exposed to high-concentration oxygen for 60 min presented with significantly impaired function of myocardial mitochondria when compared with normoxemic rats [89].

The first human trial related to the oxygen fraction in the postarrest period was published in 2006 [90]. In total, 28 patients who had witnessed OHCA were randomized to receive controlled ventilation with FiO_2_ 1.0 or 0.3, respectively, after the return of spontaneous circulation. Functional neurological status and biochemical markers of neuronal injury (neuron specific enolase—NSE, protein S100) were measured for up to 48 h after ROSC. There was a higher level of NSE at 24 h in the hyperoxemic group without any difference in mortality or neurological outcome. However, this study was significantly underpowered to detect changes in neurological status between the groups.

More human data comes from a retrospective analysis of 6,326 patients hospitalized in the ICU after CPR for cardiac arrest [10]. These persons were divided into the three groups—hypoxemia (PaO_2_ less than 8.0 kPa), normoxemia (PaO_2_ between 8.0 and 40 kPa), and hyperoxemia (PaO_2_ more than 40 kPa). A significantly higher hospital mortality (63%) was demonstrated in the hyperoxemic patients, whilst the lowest mortality was seen in normoxemic victims (44%). Hyperoxemic patients also had the highest incidence of neurological deficit at hospital discharge.

However, these findings were questioned by two recent clinical studies [91, 92]. In total, 12,108 patients resuscitated from nontraumatic cardiac arrest were divided into three groups according to their PaO_2_. The authors studied the outcomes of resuscitated adult patients divided into three groups according to their worst PaO_2_ within 24 h after CPR. The hyperoxemic group showed slightly lower survival rate than normoxemic victims but after adjustments and Cox modelling the differences became statistically insignificant [91]. Spindelboeck and colleagues studied the outcome of resuscitated adult patients divided into three groups according to their PaO_2_ at 60 min after commencing CPR. Hyperoxemic group showed higher survival rates to the hospital admission than normoxemic and hypoxemic groups but differences in neurologically intact survival rates were insignificant between the groups [92].

Another study explored the time frame of exposure to hyperoxemia after cardiac arrest [93]. The authors found that most patients were exposed to high values of PaO_2_ in the immediate period after ROSC or during the following 24 hours, suggesting that the highest hyperoxemic values are associated with treatment in the prehospital phase and the Emergency Department. Furthermore, patients after OHCA had a higher incidence of hyperoxemia than patients after in-hospital cardiac arrest. As in other published studies, there have been extensive discussions over how to define hyperoxemia.

Based on this evidence, a lower targeted oxygen therapy (spO_2_ or saO_2_ between 94 and 98%) may be beneficial in the period after ROSC. Both resuscitation guidelines published recently—the American Heart Association (AHA) guidelines and European Resuscitation Council guidelines since 2010—highlight the harmful effect of hyperoxemia after ROSC. They recommend consideration of a normoxemic strategy controlled by spO_2_ (94–98%) or saO_2_ monitoring [6, 7].

### 3.2. Ventilation and Carbon Dioxide Tension after ROSC

Controlled ventilation affects carbon dioxide (CO_2_) tension in the vascular system. Cardiac arrest is typically associated with profound metabolic acidosis. Previously, strategies have recommended hyperventilation after ROSC with the aim of decreasing PaCO_2_ and thus stabilizing the pH of arterial blood. However, a deleterious effect on brain circulation is seen if hyperventilation results in hypocapnia [94]. Cerebral hyperperfusion occurs immediately after ROSC and can persist for up to 30 minutes. The subsequent period is characterized by significantly reduced cerebral blood flow. Hypocapnia during this period potentiates vasoconstriction, which can further aggravate postresuscitation hypoxic brain injury [7]. On the other hand, insufficient CO_2_ removal is associated with hypercapnia contributing to the vasodilation of cerebral vessels and elevated intracranial pressure (ICP).

Falkenbach et al. highlighted the effect of postresuscitation therapeutic hypothermia on PaCO_2_ level [95]. Hypothermia decreases metabolic rate and carbon dioxide production. In their multicenter study, approximately 45% of patients experienced hypocapnia or hypercapnia, both of which negatively affect brain perfusion. The results of this study support the necessity of frequent and regular optimization of ventilator settings in the first 48 hours after OHCA. A number of studies have evaluated the effects of hypercapnia and hypocapnia on outcomes in adult patients after cardiac arrest. The database of the Australian and New Zealand Intensive Care Society (16,542 patients) has shown higher mortality and lower discharge rates in hypocapnic patients when compared with normocapnic and hypercapnic victims [96]. Roberts and colleagues analyzed adult cardiac arrest registry data from 193 victims and found that 69% of them experienced pathological values of PaCO_2_ after OHCA [97]. Both hypocapnia and hypercapnia were associated with worsened neurological outcome. Lee et al. studied the relationship between blood gas tensions and outcome in 213 patients after OHCA treated with therapeutic hypothermia [98]. Hypocapnia was associated with higher in-hospital mortality; both hyperoxemia and hypoxemia were associated with worsened neurological outcome.

ERC guidelines suggest maintaining normocapnia during postresuscitation care [6]. AHA 2010 guidelines recommend monitoring of CO_2_ tension with capnography and arterial blood gas analysis and keeping its level within the physiological range (PaCO_2_ 40–45 mmHg; PetCO_2_ 35–40 mmHg) [7].

## 4. Conclusions

During last decade, many papers have evaluated the issues of respiration, oxygenation, and airway management in OHCA. Some of them have been high-quality randomized controlled trials, moving the science of resuscitation forward and changing established algorithms and resuscitation protocols. The main finding arising from these studies is that, in OHCA of cardiac origin in adults, significantly interrupting chest compressions for the purposes of advanced airway management have a negative impact on patient survival and neurological outcome [36, 99]. These findings have prompted changes in BLS of adult patients with OHCA of nontraumatic origin. CAB (circulation-airway-breathing) has evolved from ABC [100] with the development of a new resuscitation philosophy—cardiocerebral resuscitation (CCR) [101, 102].

Passive oxygenation has its advocates, but one can object that its main beneficial effect is actually in minimizing the interruption of chest compressions in comparison with advanced airway management techniques.

The role of extracorporeal techniques on survival in patients after OHCA remains unclear. A few studies have demonstrated its benefit in patients with persisting cardiac arrest and with reactive pupils, though other trials have failed to report any significant benefit with this technique.

The choice of airway management technique in OHCA remains controversial. Although bag-valve mask ventilation has been repeatedly associated with better survival—including better neurological function than advanced techniques of airway management, the risk of regurgitation and aspiration cannot be underestimated. Tracheal intubation in the hands of experienced operators is still a reliable method. The evidence would suggest, however, that it should not be employed by individuals with low skills, limited experience, or infrequent exposure [37, 103]. The insertion of a supraglottic airway device in OHCA is probably associated with worse patient outcomes than other methods of airway management.

A few studies have explored the harmful effects of hyperoxemia, hyperventilation, and excessive chest inflation on patient outcome following OHCA. A significant number of these studies were performed on animal models with a small number of probands, and their interpolation to humans is difficult [104, 105]. Initial studies related to the use of an impedance threshold device (ITD), which protects against lung hyperinflation and helps to create a negative intrathoracic pressure, were promising in patients with OHCA of cardiac origin [73]. Unfortunately, a recent large trial did not show any beneficial effect of ITD on long-term survival with good neurological function [81].

The deleterious effects of pathological PaCO_2_ values after ROSC on neurological outcome have been repeatedly described. Dyscarbia is a very common finding during therapeutic hypothermia in the postresuscitation period due to the decreased metabolic demand of patients [95]. Clinicians should maintain PaCO_2_ in the upper physiological values after ROSC.

## Abbreviations

AHA: American Heart Association
ALS: Advanced life support
ATP: Adenosine triphosphate
BLS: Basic life support
BMV: Bag-valve mask ventilation
CCR: Cardiocerebral resuscitation
CFIO: Constant flow insufflation of oxygen
COCPR: Chest compression-only cardiopulmonary resuscitation
CPR: Cardiopulmonary resuscitation
DO_2_: Oxygen delivery
ECMO: Extracorporeal membrane oxygenation
ERC: European Resuscitation Council
ICU: Intensive care unit
IPPV: Intermittent positive pressure ventilation
ITD: Impedance threshold device
LMA: Laryngeal mask airway
NSE: Neuron-specific enolase
OHCA: Out-of-hospital cardiac arrest
ROS: Reactive oxygen species
ROSC: Restoration of spontaneous circulation
SAD: Supraglottic airway device
VF: Ventricular fibrillation
VT: Ventricular tachycardia.
